# Targeting Pyroptotic Cell Death Pathways in Retinal Disease

**DOI:** 10.3389/fmed.2021.802063

**Published:** 2022-01-03

**Authors:** Mary Zhao, Siqi Li, Joanne A. Matsubara

**Affiliations:** Department of Ophthalmology and Visual Sciences, Eye Care Centre, Faculty of Medicine, University of British Columbia, Vancouver, BC, Canada

**Keywords:** pyroptosis, cell death, NLRP3, caspase-1, GSDMD, age-related macular degeneration, diabetic retinopathy, glaucoma

## Abstract

Pyroptosis is a gasdermin-mediated, pro-inflammatory form of cell death distinct from apoptosis. In recent years, increasing attention has shifted toward pyroptosis as more studies demonstrate its involvement in diverse inflammatory disease states, including retinal diseases. This review discusses how currently known pyroptotic cell death pathways have been implicated in models of age-related macular degeneration, diabetic retinopathy, and glaucoma. We also identify potential future therapeutic strategies for these retinopathies that target drivers of pyroptotic cell death. Presently, the drivers of pyroptosis that have been studied the most in retinal cells are the nucleotide-binding and oligomerization domain-like receptor family pyrin domain-containing 3 (NLRP3) inflammasome, caspase-1, and gasdermin D (GSDMD). Targeting these proteins may help us develop new drug therapies, or supplement existing therapies, in the treatment of retinal diseases. As novel mechanisms of pyroptosis come to light, including those involving other inflammatory caspases and members of the gasdermin protein family, more targets for pyroptosis-mediated therapies in retinal disease can be explored.

## Introduction

Cell death has long been a subject of interest in the study of retinal pathology. The role of programmed cell death (PCD) in retinal diseases is a particularly exciting avenue of research as the regulated nature of these cell death pathways implies that they can potentially be interrupted or manipulated by pharmacological interventions. Traditionally, apoptosis was equated to PCD because it was the most well-studied and well-characterized form of PCD. Research from the 1990's suggested that apoptosis was the main mechanism of regulated cell loss in retinal degeneration and this remained a popular view for most of the 21^st^ century (1–3). The Nomenclature Committee on Cell Death now recognizes that there are other types of regulated cell death besides apoptosis–including necroptosis, ferroptosis, and pyroptosis (4). Necroptosis involves the activation of the pseudokinase mixed-lineage kinase domain-like protein (MLKL), receptor-interacting protein kinase 1 (RIPK1), and receptor-interacting protein kinase 3 (RIPK3) (5). Ferroptosis, as the name suggests, is iron-dependent and is driven by severe lipid peroxidation that results from loss of activity of the lipid repair enzyme glutathione peroxidase 4 (GPX4) (6). Finally, pyroptosis is characterized by membrane pore formation and rapid plasma membrane rupture caused by the binding of the N-terminal of gasdermin proteins to the inner leaflet of the plasma membrane (7–9). As the mechanisms of these cell death pathways have become clearer, more research has emerged supporting their involvement in retinal disease. A recent review summarized the role of necroptosis and ferroptosis in blinding eye disease (10); however, no such a review exists for pyroptosis. Here, we discuss the current understanding of pyroptosis, the research implicating pyroptotic cell death pathways in retinal diseases, and how this knowledge can be applied to identifying novel therapeutic approaches to retinopathies. Specifically, we will focus on the role of pyroptosis in the pathogenesis and potential treatment of three of the most prevalent retinal diseases–age-related macular degeneration, diabetic retinopathy, and glaucoma.

## Overview of Pyroptosis

The term “pyroptosis” was first coined in 2001 by Cookson and Brennan from the Greek roots “pyro,” relating to fire or fever, and “ptosis” meaning falling, to describe pro-inflammatory PCD (11). This distinguished pyroptosis from apoptosis, which is non-inflammatory PCD. Initially, caspase-1 was believed to be the effector of pyroptosis after *Salmonella*-infected macrophages were found to undergo a caspase-1-dependent form of cell death that was associated with pore formation and was distinguishable from apoptosis (11–13). Later, this role shifted to gasdermin D (GSDMD) when it was discovered that the cleaved, N-terminal of GSDMD (GSDMD-N) could bind to and form pores in the cell membrane, leading to pyroptotic cell death (14). Interestingly, pore-forming activity is not exclusive to GSDMD-N; in fact, most gasdermins have an N-terminal pore-forming domain and have the ability to induce pyroptosis (7). This has led to our current understanding of pyroptosis as gasdermin-mediated cell death.

The gasdermin protein family includes gasdermin A/B/C/D/E (GSDMA/B/C/D/E) and DFNB59 (Pejvakin, PJVK) in humans. Gasdermin proteins share 45% sequence homology, and all members (except for Pejvakin) contain C-terminal and N-terminal domains (7, 15). The C-terminal domain is a repressor domain that, when linked to the N-terminal domain, auto-inhibits the N-terminal's cytotoxic activity (16, 17). Inflammatory caspases or granzymes can cleave inactive, full-length gasdermin and liberate its N-terminal domain (14, 18–20). When freed, the N-terminal can then bind to phosphoinositides or cardiolipin on the inner leaflet of the plasma membrane and form membrane pores characteristic of pyroptotic cell death. Pyroptosis is also associated with the release of pro-inflammatory cytokines IL-18 and IL-1β, through these approximately 18 nm-wide membrane pores (21). This adds an additional pathological stressor to cells that is not present with apoptotic cell death and is what earns pyroptosis its designation as a pro-inflammatory form of cell death (7, 8, 13, 15). It was previously thought that extracellular fluid also enters plasma membrane pores during pyroptosis, passively causing plasma membrane rupture (PMR) through oncotic cell swelling. However, PMR is actually an active event mediated by the cell-surface protein Ninjurin 1 (NINJ1) and has been proposed to occur after pyroptotic cell death and IL-18/IL-1β release (22).

Multiple different mechanisms can lead to gasdermin cleavage in pyroptosis. The two most well-studied mechanisms are the canonical and non-canonical inflammasome pathways. The canonical inflammasome pathway, leading to canonical pyroptosis, is mediated by caspase-1. Inflammasomes are multimeric protein complexes, composed of a central sensor protein, an adaptor protein ASC (apoptosis-associated speck-like protein containing a caspase activation and recruitment domain), and pro-caspase-1. The most well-studied sensor proteins known to assemble canonical inflammasomes are NLRP1, NLRP3, NLRC4, AIM2, and pyrin (23, 24). Other proteins such as human NLRP2 and murine NLRP6 have also been implicated in inflammasome signaling (25, 26). These proteins respond to pathogen-associated and danger-associated molecular patterns (PAMPs and DAMPs), which causes “activation” (i.e., assembly) of the inflammasome. Pro-caspase-1 within the activated inflammasome undergoes autocatalytic cleavage into mature caspase-1, and mature caspase-1 can then cleave GSDMD to cause pyroptosis (14, 23, 27). Mature caspase-1 also has the ability to convert pro-IL-18 and pro-IL-1β into their mature forms that are released from membrane pores during pyroptosis (8, 12, 13). Intriguingly, inflammasomes can also be activated and release inflammatory cytokines without necessarily causing cell death through an unknown mechanism that may involve the Toll-IL-1R protein SARM (sterile alpha and HEAT armadillo motif-containing protein) (9, 28). The non-canonical pyroptosis pathway does not depend on caspase-1; rather, it is triggered by the direct binding of procaspase-4/5 in humans, or -11 in mice, to intracellular lipopolysaccharide (LPS). Like caspase-1, activated caspase-4/5/11 can then go on to cleave GSDMD to execute pyroptosis. However, these caspases cannot directly process pro-IL-18 and pro-IL-1β into their mature forms (15, 18, 29, 30).

Up until recently, the canonical and non-canonical inflammasome pathways leading to GSDMD activation were the only known pyroptotic pathways. However, in 2017, both *in vitro* and *in vivo* studies showed that pyroptosis could be induced by GSDME expression and cleavage of GSDME into GSDME-N by caspase-3 (31, 32). Furthermore, in 2018, it was found that GSDMD could also be cleaved by caspase-8 in mouse macrophages (33, 34). These findings were especially interesting because caspases 3 and 8 were previously associated with apoptosis and were not thought to be able to interact with gasdermins. Overall, these studies improve our understanding of pyroptosis as we now know that activation of other caspases besides caspases 1/4/5/11 and other gasdermins besides GSDMD can also cause pyroptotic cell death.

## Role of Pyroptosis in Retinal Disease

Major findings from studies investigating pyroptosis in age-related macular degeneration, diabetic retinopathy, and glaucoma are summarized in Tables 1–3, respectively.

**Table 1:** Studies investigating pyroptosis in AMD.

**Study**	**Model/Cell type studied**	**Technique**	**Findings**	**Relevance of findings to pyroptosis**
Tarallo et al. (35)	Human RPE cells transfected with pAlu	MTS cell viability assay	The cytoprotective agent glycine (pyroptosis inhibitor) did not rescue *Alu* RNA-induced RPE degeneration	*Alu* RNA accumulation (a feature of GA) may not induce RPE degeneration via pyroptosis
Kerur et al. (36)	Human RPE cells transfected with pAlu, subretinal injection of pAlu in mice	Immunohistochemistry	*Gsdmd^−/−^* mice were resistant to *Alu* RNA-induced RPE degeneration; however, there was no observed cleavage of GSDMD into its N-terminal-pore-forming p30 fragment in pAlu-transfected human primary RPE cells or WT mice subretinally injected with *Alu* RNA	GSDMD is required for *Alu* RNA-induced RPE degeneration, but plays a non-pyroptotic role
Tseng et al. (37)	Lysosomal destabilization in ARPE-19 cells using Leu-Leu-OMe treatment	LDH release, caspase-1 inhibition with 10 μM Z-YVAD-FMK	Lysosomal destabilization induced LDH release from ARPE-19 cells, mediated by caspase-1	ARPE-19 cells undergo pyroptosis in response to lysosomal destabilization
Gao et al. (38)	Aβ intravitreal injections in Long-Evans rats	Western blotting	RPE-choroid protein lysates of rats receiving Aβ injections showed significantly greater cleavage of pro-GSDMD into GSDMD-N compared to controls	Aβ upregulates GSDMD-N, a driver of pyroptosis, in RPE cells
Sun et al. (39)	Aβ-treated ARPE-19 cells	Flow cytometry for positive PI and caspase-1 staining	Aβ significantly increased the proportion of ARPE-19 cells staining positive for both PI and caspase-1	Aβ induces pyroptosis in ARPE-19 cells
Yang et al. (40)	Aβ-treated ARPE-19 cells	Immunofluorescence, scanning electron microscopy	Aβ triggered increased levels of GSDMD-N, as well as swelling, bubbling, and cell membrane rupture in ARPE-19 cells	Aβ causes upregulation of a pyroptosis effector and morphological characteristics of pyroptosis in ARPE-19 cells
Liao et al. (41)	atRAL-treated ARPE-19 cells	Western blotting	Lysates of ARPE-19 cells treated with 15 uM atRAL showed increased levels of cleaved GSDME at 6 and 12 h but GSDMD remained full-length	atRAL-treated ARPE-19 cells may undergo GSDME-mediated, rather than GSDMD-mediated, pyroptosis

*RPE, retinal pigment epithelium; pAlu, plasmid coding for Alu RNA; GA, geographic atrophy; WT, wild-type; ARPE-19, human adult retinal pigment; LDH, lactate dehydrogenase; Aβ, amyloid beta; PI, propidium iodide; GSDMD/E, gasdermin D/E; GSDMD-N, N-terminal of gasdermin D; atRAL, all-trans retinal*.

**Table 2:** Studies investigating pyroptosis in DR.

**Study**	**Model/Cell type studied**	**Technique**	**Findings**	**Relevance of findings to pyroptosis**
Jiang et al. (42)	Primary human RECs incubated in 25 mM high glucose	Western blotting	Increased protein levels of NLRP3, cleaved caspase-1, and IL-1β in high glucose vs. normal glucose group	The NLRP3/caspase-1-mediated pyroptotic pathway may be activated in HRMECs in response to high glucose
Chen et al. (43)	HRMECs incubated in 30 mM high glucose	Western blotting	Increased protein levels of NLRP3, cleaved caspase-1, and IL-1β in high glucose vs. normal glucose group	The NLRP3/caspase-1-mediated pathway may be activated in RECs in response to high glucose
Gu et al. (44)	HRMECs incubated in 30 mM high glucose	PI and caspase-1 FLICA staining, flow cytometry	Pyroptosis and caspase-1 activity were markedly increased in high-glucose-treated HRMECs vs. the control group	High glucose promotes pyroptotic cell death in HRMECs
Gan et al. (45)	HRPs incubated in 30 mM high glucose	Pore formation: PI uptake Cell lysis: LDH release Cytokine release: ELISA	High glucose-treated HRPs experienced greater pore-formation, cell lysis, and release of IL-1β and IL-18 compared to controls → these effects were reversed with NLRP3, caspase-1, or GSDMD inhibition	High glucose can induce the loss of HRPs via GSDMD-mediated pyroptosis
Yu et al. (46)	HRPs incubated in 200 μg/ml AGE-BSA	Protein expression: Western blotting Cytokine release: ELISA LDH activity: LDH assay kit Cell viability: cell counting kits	AGE-BSA increased expression of active caspase-1 and GSDMD-N and promoted secretion of IL-1β, IL-18, and LDH in HRPs, alongside decreasing HRP viability	HRPs undergo GSDMD-mediated pyroptosis when treated with AGE-BSA
Du et al. (47)	Mouse primary retinal Müller cells incubated in 30 mM high glucose	Western blotting	Increased levels of NLRP3, cleaved caspase-1, and IL-1β in high glucose-treated mouse retinal Müller cells	The NLRP3/caspase-1-mediated pyroptotic pathway may be activated in Müller cells cultured under high glucose conditions
Xi et al. (48)	ARPE-19 cells incubated in 50 mM high glucose	Western blotting	High glucose upregulated protein expression of caspase-1, GSDMD, NLRP3, IL-1β, and IL-18 in ARPE-19 cells	High glucose may promote GSDMD-mediated pyroptosis in ARPE-19 cells
Zha et al. (49)	ARPE-19 cells incubated in 50 mM high glucose	Western blotting	High glucose upregulated protein expression of caspase-1, GSDMD, NLRP3, IL-1β, and IL-18 in ARPE-19 cells	High glucose may promote GSDMD-mediated pyroptosis in ARPE-19 cells

*HRMECs, human retinal microvascular endothelial cells; RECs, retinal endothelial cells; PI, propidium iodide; FLICA, fluorochrome-labeled inhibitors of caspases; HRPs, human retinal pericytes; LDH, lactate dehydrogenase; ELISA, enzyme-liked immunosorbent assay; AGE-BSA, advanced glycation end-product modified bovine serum albumin; ARPE-19, human adult retinal pigment epithelial cell line-19; NLRP3, nucleotide-binding and oligomerization domain (NOD)-like receptor family pyrin domain-containing 3; IL-1β; interleukin-1β; IL-18, interleukin-18; GSDMD, gasdermin D; GSDMD-N, N-terminal of gasdermin D*.

**Table 3:** Studies investigating pyroptosis in glaucoma.

**Study**	**Model/Cell type studied**	**Technique**	**Findings**	**Relevance of findings to pyroptosis**
Chi et al. (50)	Mouse model of acute IOP-induced glaucoma	PCR, western blotting	TLR4 deficiency protected against inflammasome activation and RGC death after acute IOP elevation via caspase-1 and caspase-8-dependent pathways	Pyroptosis-associated inflammatory pathways take place and cause RGC death after acute IOP elevation
Chi et al. (51)	Mouse model of acute IOP-induced glaucoma	PCR, western blotting	Inhibition of HMGB1, like TLR4 deficiency, protected against inflammasome activation and RGC death after acute IOP elevation via caspase-1 and caspase-8-dependent pathways	Pyroptosis-associated inflammatory pathways take place and cause RGC death after acute IOP elevation
Qi et al. (52)	RIR injury rat model	TUNEL staining, western blotting	Inhibition of TLR4 increased RGC survival by decreasing apoptosis	TLR4-mediated pathway may lead to RGC apoptosis rather than pyroptosis after RIR injury
Pronin et al. (53)	Acute OHT mouse model	Western blotting, immunohistochemistry	Within a few hours of inducing acute OHT in mouse eyes, retinal levels of GSDMD, caspase-1, and NLRP3 were significantly increased	Markers of GSDMD-mediated pyroptosis are upregulated in the retina after exposure to acute OHT
Chen et al. (54)	RIR injury mouse model	HE staining, retrograde FG-labeled imaging, immunofluorescence, western blotting	Genetic deletion of *GSDMD* significantly increased retinal thickness and decreased RGC death after RIR injury	Absence of an effector of pyroptosis protects against RGC death after RIR injury
Dong et al. (55)	Chronic OHT rat model	Western blotting	Protein levels of mature caspase-1 were elevated in rat retinas after chronic OHT	Caspase-1 processing, which can lead to pyroptosis, is increased in rat retinas with chronic OHT
Zhang et al. (56)	Chronic OHT mouse model	Western blotting	Protein levels of NLRP3 and cleaved caspase-1 were elevated during the chronic OHT process	Components of the canonical pyroptotic pathway are activated in a chronic OHT mouse model
Wan et al. (57)	RIR injury mouse model	Western blotting	RIR injury increased GSDMD-N expression in Iba-1+ microglia	An effector of pyroptosis is upregulated in microglia after RIR injury

*IOP, intra-ocular pressure; RGC, retinal ganglion cell; PCR, polymerase chain reaction; RIR, retinal ischemia/reperfusion; TUNEL, terminal deoxynucleotidyl transferase dUTP nick end labeling; OHT, ocular hypertension; HE, hematoxylin and eosin; FG, fluoro-gold; NLRP3, nucleotide-binding and oligomerization domain (NOD)-like receptor family pyrin domain-containing 3; IL-1β; interleukin-1β; IL-18, interleukin-18; GSDMD, gasdermin D; GSDMD-N, N-terminal of gasdermin D; TLR4, toll-like receptor 4; HMGB1, high-mobility group box 1*.

### Pyroptosis and Age-Related Macular Degeneration

Age-related macular degeneration (AMD) is the most common cause of irreversible vision loss among the elderly in the developed world, and is projected to affect 288 million people globally by 2040 (58). AMD is a neurodegenerative disease; accumulation of drusen deposits results in progressive degeneration of photoreceptors and retinal pigment epithelium (RPE), primarily in the macula. Clinically, AMD can present as a spectrum of disease phenotypes, with the severity of the disease depending on drusen size. Earlier stages of AMD are defined by the presence of medium-sized drusen deposits and do not present with vision loss. As drusen grow in size and number, atrophy of photoreceptors, RPE, and choriocapillaris and scotoma development can occur. These features are characteristic of a late stage of AMD called geographic atrophy (GA or “dry AMD”). Large drusen also increase the risk of developing neovascular AMD, in which new, abnormal vessels form and invade the outer retina, subretinal space, or subRPE space. Exudative or “wet AMD” occurs when these new vessels rupture and leak exudates, causing fluid accumulation/hemorrhage and severe central vision loss if left untreated (59, 60).

In 2011, Kaneko and colleagues discovered that reduction of the RNase DICER1 led to accumulation of *Alu* RNA, non-coding RNA transcripts expressed by the *Alu* retrotransposon, in RPE from human donor eyes with GA. *Alu* RNA accumulation, in turn, resulted in RPE degeneration in both humans and mice (61). A year later, the same group found that *Alu* RNA did not induce RPE degeneration in *Nlrp3*^−/−^ or *Casp1*^−/−^ mice. This suggested that the canonical NLRP3/caspase-1-dependent pyroptotic pathway may be involved in RPE degeneration in AMD. However, glycine, a cytoprotective agent that attenuates pyroptosis, did not rescue *Alu* RNA-induced RPE degeneration in the same study. The authors concluded that while NLRP3 and caspase-1 are critical for *Alu* RNA-induced RPE degeneration, *Alu* RNA does not induce RPE degeneration via pyroptosis (35). *Gsdmd*^−/−^ mice, like *Nlrp3*^−/−^ and *Casp1*^−/−^ mice, were shown to be resistant to *Alu* RNA-induced RPE degeneration in a study by Kerur et al. (36), but there was no observed cleavage of GSDMD into its N-terminal pore-forming domain. Furthermore, reconstituting *Gsdmd*^−/−^ mice with a GSDMD mutant unable to undergo cleavage (pGSDMD-D276A) restored susceptibility to *Alu* RNA-induced RPE degeneration. Full-length GSDMD cannot induce pyroptosis; thus, it must exert its effects on *Alu* RNA-induced RPE toxicity through another mechanism. Administration of mature IL-18 to *Gsdmd*^−/−^ mice restored *Alu* RNA-induced RPE degeneration and led to the appearance of annexin V positive, propidium iodide (PI) negative staining RPE cells. This suggested that GSDMD plays a role in *Alu* RNA-induced cytotoxicity via IL-18-dependent apoptosis, rather than pyroptosis, in RPE (36). While the above studies do not support that RPE cells undergo pyroptotic cell death in response to *Alu* RNA, they do identify NLRP3, caspase-1, and full-length GSDMD as potential therapeutic targets for AMD, particularly for GA.

Lysosomal destabilization, which can result from drusen accumulation and trigger inflammasome activation, has also been studied for its potential to cause pyroptosis in AMD. Lysosomal destabilization with Leu-Leu-OMe treatment induced IL-1β and LDH release from ARPE-19 cells, mediated by caspase-1. These findings indicate that lysosomal destabilization leads to a caspase-1-dependent, pro-inflammatory, and lytic form of cell death in RPE, characteristic of pyroptosis (37). Caspase-1 inhibition may also therefore be a worthwhile therapeutic strategy for AMD treatment.

Additional support for RPE pyroptosis in AMD comes from research on amyloid beta (Aβ), a component of drusen. After the NLRP3 inflammasome was activated by repeated intravitreal injections of Aβ into the eyes of Long-Evans rats, RPE-choroid protein lysates from Aβ-injected animals showed significantly increased levels of GSDMD-N and decreased levels of full-length GSDMD (38). This supported that GSDMD-mediated pyroptosis can be activated in RPE cells and that NLRP3 and GDSMD-N are possible targets for AMD therapy. In another study using Aβ-induced ARPE-19 cells as a model for AMD, Baicalin was found to alleviate Aβ-induced pyroptosis detected by flow cytometry for positive PI and caspase-1 labeling (39). The protective action of Baicalin was mediated by upregulating miRNA-223, which had been previously found to reduce the expression of NLRP3 (62). Baicalin's anti-pyroptotic effects were reversed by miRNA-223 knockdown, whereas adding MCC950 (an NLRP3 inhibitor) once again reduced pyroptosis (39). Lycium Barbarum Polysaccharides (LBP), present in Goji berries, also rescued Aβ-induced reduction of RPE cell viability at low (3.5 mg/L) and high (14 mg/L) doses via attenuation of pyroptosis. Aβ triggered increased levels of GSDMD-N and caused morphological changes in RPE characteristic of pyroptosis, both of which were reversed by LBP treatment (40). As such, inhibiting pyroptosis using Baicalin or LBP may potentially be therapeutic for AMD.

There is also evidence that GSDME-mediated, rather than GSDMD-mediated, pyroptosis occurs in RPE in the all-trans retinal (atRAL) model of AMD. atRAL is generated during the visual (retinoid) cycle and can accumulate in visual cycle anomalies, causing RPE atrophy in AMD. Cleavage of GSDME was detected at 6 and 12 h in lysates of atRAL-treated ARPE-19 cells but GSDMD remained intact, suggesting that atRAL triggers pyroptosis in ARPE-19 cells by activating the caspase-3/GSDME pathway of pyroptosis (41). Research on GSDME-mediated pyroptosis in retinal cells is sparse and further study is required to see if this pathway can be targeted for the treatment of AMD.

### Pyroptosis and Diabetic Retinopathy

Diabetic retinopathy (DR) is a leading cause of preventable vision loss in working-age adults and can be broadly classified into two clinical stages: non-proliferative diabetic retinopathy (NPDR) and proliferative diabetic retinopathy (PDR) (63). Early in NPDR, retinal pericytes that support retinal capillaries are lost, causing capillary occlusion and increased vascular permeability. On fundoscopy, intra-retinal hemorrhages, microaneurysms, and exudates called “cotton wool spots” may be observed in NPDR. NPDR can eventually lead to PDR, in which vascular endothelial growth factor (VEGF) promotes neovascularization in the retina. These newly formed vessels have leaky tight junctions that can result in vitreous hemorrhage or tractional retinal detachment (TRD), and cause vision loss. Another cause of vision loss in DR is diabetic macular edema (DME), where the macula becomes thickened due to breakdown of the blood-retina barrier (BRB) (64). DR is primarily considered a microvascular disease and breakdown of the BRB is key to this disease state. Maintenance of the BRB depends on the functioning of an interdependent network of cells–including endothelial cells that make up the inner BRB, supportive Müller cells and pericytes, and RPE cells which form the outer BRB (65).

A previous review discussed modes of retinal cell death in DR (66). Only Müller cell loss in diabetes was outlined to show characteristics of pyroptosis while other retinal cells including endothelial cells and pericytes were thought to primarily undergo apoptosis or necrosis. More recent studies have found signs of possible pyroptotic cell death in many types of retinal cells in DR models including endothelial cells, pericytes, Müller cells, and RPE. Endothelial cells line the retinal microvasculature and comprise the highly selective inner BRB (65). NLRP3/caspase-1 activation and IL-1β release have been recorded in retinal endothelial cells (RECs) in various *in vivo* and *in vitro* models of DR (42, 43, 67). Pyroptotic cell death and caspase-1 activity were markedly increased in human retinal microvascular endothelial cells (HRMECs) incubated in 30 mM high glucose compared to controls (44). Pyroptosis was identified in this study using PI/caspase-1 fluorochrome inhibitor (FLICA) staining and flow cytometry. This suggests that canonical pyroptosis may take place in RECs and targeting NLRP3 and caspase-1 may be a treatment strategy to prevent their loss in DR. Retinal pericytes provide structural support to retinal vessel walls and regulate the expression of tight junctions in adjacent endothelial cells (65). A study published in 2020 showed that silencing GSDMD inhibits IL-1β and IL-18 release, decreases pore formation, and decreases lysis of human retinal pericytes exposed to 30 mM high glucose (45). Another study using advanced glycation endproducts modified bovine serum albumin (AGE-BSA) to simulate the DR environment found increased expression of active caspase-1 and GSDMD-N as well as increased secretion of IL-1β, IL-18, and lactate dehydrogenase (LDH) in human retinal pericytes (HRPCs) alongside decreased HRPC viability (46). Thus, pyroptotic pericyte loss may occur in DR and blocking caspase-1 and GSDMD can potentially preserve pericyte viability. Müller cells are the principal glial cells of the retina and, because of their innate role in mediating neuroinflammation, have long been speculated to participate in pyroptosis (66). Protein levels of NLRP3, ASC, cleaved caspase-1 and cleaved IL-1β were increased by 30 mM high glucose in mouse primary retinal Müller cells. Furthermore, NLRP3 antagonism with the inhibitor drug MCC950 downregulated high glucose-induced upregulation of pro-angiogenic factors including VEGF (47). This implicated activation of the NLRP3 inflammasome pathway in Müller cells in DR and provided support that NLRP3 specifically plays a role in late-stage neovascularization. Finally, while the RPE (part of the outer BRB) is not traditionally viewed to play a central role in the pathophysiology of DR, ARPE-19 cells have recently been found to undergo pyroptotic cell death under stimulation with 50mM glucose, which increased expression of pyroptosis-associated proteins NLRP3, caspase-1, and GSDMD (48, 49). Overall, the NLRP3/caspase-1/GSDMD canonical pyroptotic pathway appears to play a role in the loss of endothelial cells, pericytes, Müller cells, and RPE in cell culture, and in animal and human models for DR. However, few studies have directly demonstrated that the effector of pyroptosis, GSDMD-N, is activated in DR. Future studies aimed at GSDMD-N are needed to evaluate its potential to be a target for DR therapy.

### Pyroptosis and Glaucoma

Glaucoma is a group of ocular diseases characterized by the progressive loss of retinal ganglion cells (RGCs), the neurons that communicate visual information from the retina to the brain (68). It is another leading cause of irreversible blindness worldwide and is projected to affect 112 million individuals aged 40–80 by 2040 (69). Various risk factors for glaucoma have been identified–the most notable being elevated intraocular pressure (IOP) and age–but the exact molecular mechanisms that link these risk factors to RGC loss are still under investigation. Past research has demonstrated that RGCs die by apoptosis (70). However, recent studies have implicated inflammasomes, caspase-1, and GSDMD in acute and chronic models of glaucoma, suggesting that apoptosis is not the only form of cell death involved in glaucomatous RGC loss.

In a mouse model for acute elevated IOP-induced glaucoma, NLRP1, NLRP3, ASC, and caspase-1 levels were rapidly upregulated in the retina after ischemic injury. Knockdown of the gene encoding toll-like receptor 4 (TLR4) using *TLR4*^−/−^ mice reduced inflammasome production and RGC death after ischemic injury (50). TLR4 deficiency therefore seems to protect against RGC death through the inactivation of canonical inflammasomes and may be a potential treatment strategy for acute glaucoma. Chi et al. (50) also showed that caspase-8 is the link between TLR4 and NLRP1/NLRP3 activation. As discussed previously, caspase-8 is traditionally thought of as an initiator of apoptosis but has also been found to play non-apoptotic roles (71). In support of this idea, this study demonstrated that inhibition of caspase-8 significantly reduced levels of NLRP1, NLRP3, ASC, caspase-1, and IL-1β, and also attenuated IOP-induced RGC death. Interestingly, inhibition of caspase-8 completely suppressed production of IL-1β while inhibition of caspase-1 only partially suppressed production of IL-1β. Therefore, therapeutic strategies targeting caspase-8 may be more effective at preventing inflammation in acute glaucoma than those targeting caspase-1. A year later, the same authors found that high-mobility group box 1 (HMGB1), an endogenous ligand of TLR4, is also involved in the above pathway. Inhibition of HMGB1 suppressed the production of NLRP3, ASC, activated caspase-1, activated caspase-8, and IL-1β, and also decreased RGC death after acute IOP elevation similarly to TLR4 (51). Thus, blocking HMGB1 is another way to target TLR4-induced inflammasome pathways in the treatment of acute glaucoma. A study using a retinal ischemia/reperfusion (RIR) injury rat model provided further support for TLR4-induced activation of NLRP3 and also found that inhibition of TLR4 decreased loss of RGCs. However, the type of cell death studied and detected to occur in these RGCs was apoptosis rather than pyroptosis (52). Aside from acute ischemic injury, inflammasomes are also involved in RGC loss from optic nerve crush injury. Following partial optic nerve crush (pONC) in mice, NLRP3 was upregulated at the site of injury and then propagated to the optic nerve head (ONH) and the entire retina within 1 day. Furthermore, *NLRP*3^−/−^ mice experienced delayed RGC somal loss for 1 week and similarly delayed/decreased axon loss (72). These findings are in congruence with those from previous studies and support that NLRP3 is important for inflammation and RGC death in models of acute glaucoma, making it a worthwhile target for therapy.

The above studies implicated drivers of canonical pyroptosis in glaucomatous RGC death, but they did not study the effector of canonical pyroptosis, GSDMD. Pronin et al. (53) found that within a few hours of inducing acute ocular hypertension (OHT) in mouse eyes, retinal levels of GSDMD, in addition to activated caspase-1 and NLRP3, were significantly increased. RGCs were also shown to be the first cell type in the ganglion cell layer (GCL) to significantly express cleaved GSDMD after acute OHT injury (53). Taken together, these findings suggest that after acute elevation of IOP, inflammasomes are activated in the retina and caspase-1 cleaves GSDMD to potentially trigger pyroptosis in RGCs. In a mouse RIR injury model, intravitreal injection of a *Casp1* inhibitor (Z-YVAD-fmk) markedly reduced cleavage of IL-1β and GSDMD, and restored RGC numbers during RIR injury. Furthermore, genetic deletion of *GSDMD* significantly increased retinal thickness and decreased RGC death after RIR injury (54). Therefore, both caspase-1 inhibition and knockdown of GSDMD expression are possible strategies to therapeutically attenuate RGC death in acute glaucoma. This study also reconciled the previously discovered role of caspase-8 in elevated IOP-induced RGC death with findings from other disease states that caspase-8 can cleave GSDMD, by showing that *Casp8* silencing in mice significantly lowered levels of cleaved GSDMD protein after RIR injury (refer to Supplementary Figure 1 for a summary of proposed caspase-8-mediated apoptotic and pyroptotic pathways in acute glaucoma) (33, 34, 54).

The bulk of the research on pyroptosis in glaucoma has been done on acute models, as outlined above. Few studies have looked at the role of pyroptosis in chronic glaucoma. In human donor eyes of chronic glaucoma patients, various inflammasome components, including NLRP3 and caspase-1, were found to be upregulated along with significant cleaved caspase-1 expression in the retina. These early findings suggested that caspase-1 is activated by inflammasome assembly in chronic glaucomatous human retinas (73). NLRP3 and cleaved caspase-1 protein levels were also elevated in the retina of rodent models of chronic OHT (55, 56). These studies implicated P2X7 as the upstream activator of NLRP3. P2X7 receptors are nonselective cation channel receptors that contribute to inflammation in the central nervous system and are activated by ATP (74). Activation of the P2X7 receptor with an agonist (BzATP) increased expression of *NLRP3, Casp-1*, and *ASC* in rat retinal microglia. Inhibition of the above pathway using the P2X7 inhibitor A438079 or the NLRP3 inhibitor MCC950 decreased microglial activation and protected against RGC death (56). Thus, inhibiting the P2X7-NLRP3 pathway may be a therapeutic strategy for reducing microglial activation and subsequent RGC death in chronic glaucoma. Research on inflammatory signaling in glaucoma pathogenesis has also identified other ion channels located at the surface of RGCs, such as Transient Receptor Potential Vanilloid isoform 4 (TRPV4) and Pannexin-1 (Panx1), that act as potential sensors and effectors of mechanical strain, ischemia, and inflammatory responses. These signaling pathways are also associated with RGC axonal injury and cell death and can be further explored for potential interactions with inflammasome pathways in chronic glaucoma (75).

NLRP3 and caspase-1 were shown to be increased in the retina in the chronic glaucoma models above. However, whether these pyroptosis inducers and the pyroptosis effector GSDMD are expressed in neurons and RGCs specifically is still debated. There is evidence from other disease states that neurons express NLRP3. Functional inflammasomes and caspase-1 activity were present in cultured human CNS neurons and NLRP3 expression was detected in mesencephalic neurons in a Parkinson's disease model (76–78). However, in glaucomatous human donor eyes, cleaved caspase-1 was more prominent in non-ganglion cells (Brn-3-negative glial cells) (73). This favored that RGCs may undergo cell death through a glial-cell mediated inflammatory pathway. Pronin et al. (53), discussed above, demonstrated the upregulation of NLRP3 inflammasome in RGCs and astrocytes in acute OHT. Zhang et al. (56) from above also supported a glial cell-mediated inflammatory pathway by showing increased expression of inflammasome components in rat retinal microglia rather than RGCs. Our recent research using chronic glaucoma mouse model DBA/2J demonstrated an age-dependent upregulation of NLRP3 in RGCs and a concomitant increase in intraocular pressure (79). RIR injury in mice increased GSDMD-N expression in Iba-1+ microglia, suggesting that microglia undergo pyroptosis in response to RIR injury. On the other hand, RGCs in this study were found to undergo apoptosis (57). All in all, these controversies suggest that inflammation in the glaucomatous eye consists of multiple levels of responses that, at present, we do not fully understand. Neuronal cells including RGCs, possibly perturbed by age-related and/or IOP-induced inflammatory stress, activate glial cells by releasing DAMPs and PAMPs, which could further result in the release of pro-inflammatory cytokines and contribute to neurotoxicity and loss of RGCs. Alternatively, sensors on RGCs may respond to ischemia and inflammatory stress and lead to the remodeling of axons and cell death (50, 80). More research is needed, particularly in chronic models of glaucoma, to determine how pyroptosis fits into this inflammatory picture and whether pyroptotic drivers are appropriate therapeutic targets for glaucoma.

## Impact of Targeting Pyroptotic Cell Death Pathways on Existing Therapeutic Strategies for Retinal Diseases

Potential novel therapeutic targets for AMD, DR, and glaucoma have been highlighted throughout this review and are summarized in Table 4. In brief, majority of the suggested strategies target the canonical, NLRP3/caspase-1/GSDMD-mediated pyroptotic cell death pathway. A few studies also supported targeting caspase-3/GSDME and caspase-8/GSDMD pathways in AMD and glaucoma. Aside from the possibility of using these targets to develop new drugs for retinal diseases, targeting pyroptotic cell death pathways can also have an impact on existing therapies for retinal diseases, namely anti-VEGF. Anti-VEGF therapy is the mainstay of treatment for ocular angiogenic disease processes including AMD and DR (81). Studies have shown that NLRP3 inflammasome-mediated pathways can also affect angiogenesis in AMD and DR, and this evidence will be reviewed below. Targeting these pathways may be an alternative strategy to anti-VEGF treatment or enhance the therapeutic effect of existing anti-VEGF treatments.

**Table 4:** Potential pyroptotic targets for AMD, DR, and glaucoma therapy.

	**Potential pyroptotic targets for therapy**					
**Disease**	**NLRP3**	**Caspase-1**	**Caspase-3**	**Caspase-8**	**GSDMD, GSDMD-N**	**GSDME, GSDME-N**
AMD	✓	✓	✓		✓	✓
DR	✓	✓			✓	
Acute glaucoma	✓	✓		✓	✓	
Chronic glaucoma	✓	✓				

*✓represents that the pyroptosis-related protein has been demonstrated to play a role in the retinal disease on the left and may therefore be a potential novel therapeutic target for that disease. AMD, age-related macular degeneration; DR, diabetic retinopathy; NLRP3, nucleotide-binding and oligomerization domain (NOD)-like receptor family pyrin domain-containing 3; GSDMD/E-N, gasdermin-D/E; GSDMD-N, N-terminal of gasdermin D/E*.

We know that inflammasome activation and release of inflammatory cytokines are associated with pyroptotic cell death; thus, we may expect that inhibiting these factors would have a protective effect in retinal disease. On the contrary, *Nlrp3*^−/−^ and *IL-18*^−/−^ mice showed significantly more choroidal neovascularization (CNV) development and subretinal hemorrhage compared to wild-type (WT) mice in a laser-induced model of wet AMD. Furthermore, intravitreal injections of IL-18-neutralizing antibodies after laser-induced CNV resulted in significantly increased CNV development in WT mice, suggesting that IL-18 may protect against CNV through the downregulation of VEGF. Indeed, treatment with IL-18 significantly decreased the amount of VEGF secreted by human ARPE-19 cells as well as mouse brain microvascular endothelial cells (82). Therefore, NLRP3 could be used as a protective agent against AMD and delivering IL-18 to the eye may have a therapeutic effect on CNV progression by decreasing VEGF. The latter was also supported by another study that found that deficiency of IL-18 significantly increased the number of CNV lesions in VEGF-A^hyper^ mice (83). In 2014, Doyle et al. (84) further demonstrated that IL-18 injection would be safe to use in human eyes. They did not find any measurable cell death, changes in cell morphology, or compromise of plasma membrane integrity even when hyper-physiological doses of recombinant human IL-18 were applied to human ARPE-19 cells and native human RPE cells from three donors. Interestingly, Doyle et al. (84) also showed that IL-18 could enhance the CNV-attenuating effects of anti-VEGF therapy when applied in tandem as an intravitreal injection or systemically via a single subcutaneous dose. CNV volume was most significantly reduced when intravitreal injection of DMS1529 (mouse anti-VEGF) was combined with either intravitreal or subcutaneous administration of GSK (mouse IL-18) in C57BL/6J mice after laser-induced CNV. Systemic IL-18 therapy was also effective at reducing CNV volume alone–subcutaneous administration of GSK at a dose of 0.1 or 1.0 mg/kg 1 day before, and on each day after, laser-induced CNV both significantly attenuated CNV and CNV-induced permeability with no observable adverse effects (84). This shows the potential of using intravitreal or subcutaneous IL-18 separately or as an adjunct to existing anti-VEGF therapies to treat wet AMD pathology.

While the above research is promising, it has been met with some controversy. Tarallo et al. (35) found the opposite effect of IL-18 where IL-18 neutralization protected against RPE death in a mouse model for GA and IL-18 levels were significantly greater in human eyes with GA than in healthy controls. This implies that IL-18 is cytotoxic and may signify that IL-18 plays different roles in wet vs. dry AMD. IL-18 levels were also found to be significantly elevated in the serum of AMD patients compared to healthy controls, suggesting that higher systemic levels of IL-18 are associated with AMD diagnosis (85). This opposes the above suggestion that systemic injection of IL-18 can be therapeutic for AMD. Furthermore, while the studies by Doyle and colleagues suggested that NLRP3 could be used as a protective agent in AMD, other studies have found that NLRP3 activation/consequent increase in active IL-1β is pro-angiogenic and promotes VEGF-induced AMD pathologies (83). Nucleoside reverse transcriptase inhibitors (NRTIs) such as stavudine (d4T) also reduced CNV volume in a laser-induced mouse model of CNV via blockade of a P2X7-induced pathway of inflammasome activation (86). In DR, studies have proposed that the pro-inflammatory events associated with NLRP3 activity cause breakdown of the BRB and subsequent neovascular response leading to PDR (87). Inhibition of caspase-1 with minocycline prevented acellular capillary development in STZ-induced diabetic and galactosemia mouse models (88). Elevated protein expression of NLRP3, caspase-1, and inflammatory cytokines was found in the proliferative membranes of human donor eyes with PDR compared to healthy controls (89). Similar results were seen in vitreous fluid samples of DR patients, especially in PDR eyes with TRD and active neovessel formation (90, 91). Finally, NLRP3 inhibition with MCC950 downregulated high glucose-induced upregulation of pro-angiogenic factors including VEGF (47). In sum, NLRP3-mediated inflammatory pathways are involved in angiogenic disease processes in AMD and DR, but further research is required to resolve the debate over whether its role is deleterious or beneficial.

## Discussion

In this review, we have outlined the role of pyroptosis as a gasdermin-mediated inflammatory form of PCD in three common retinal diseases–age-related macular degeneration, diabetic retinopathy, and glaucoma. In AMD, GSDMD-mediated pyroptosis appears to occur in RPE when triggered by lysosomal destabilization or Aβ while GSDME-mediated pyroptosis occurs in the atRAL model of AMD. The research on pyroptosis in DR is in more preliminary stages, with most of its evidence for pyroptosis being limited to inflammasome activation rather than gasdermin activation in endothelial cells, pericytes, Müller cells, and RPE. Finally, there is support for gasdermin involvement in RGC loss in acute glaucoma, but evidence in chronic glaucoma models remains in its infancy. All in all, as our understanding of pyroptosis has grown and evolved, there is more support for its involvement in retinal disease. However, there are still many limitations in our understanding of pyroptosis in retinal disease that must be addressed.

Firstly, the involvement of pyroptosis in retinal disease does not exclude the occurrence of other forms of PCD such as apoptosis, ferroptosis, and necroptosis. There is substantial evidence for the involvement of these other PCD pathways in retinal disease as well (10, 66, 92). Further research is needed to uncover how different forms of PCD interact with each other in the retina and what factors ultimately determine the type of PCD an individual retinal cell will succumb to in pathological states. This information is vital in the development of therapies targeting PCD. If we target a form of PCD that is not the primary mode of cell death naturally occurring in AMD, DR, or glaucoma, then such treatments for these diseases may be ineffective. Or, if blocking one form of PCD such as apoptosis causes another, more inflammatory cell death mechanism to occur, we could potentially do more harm. A limited number of studies have provided some insight into how different types of PCD may be linked. Jiang et al. (93) showed that the caspase-3/GSDME pathway can result in either apoptosis or pyroptosis, depending on the expression level of GSDME. GSDME may therefore be the link between PCD pathways we have been looking for and provide an explanation for why we have been able to identify both apoptotic and pyroptotic mechanisms in retinal disease. On the other hand, Kayagaki et al. (22) identified that NINJ1 plays a potent role in causing plasma membrane rupture and DAMP-release not only following pyroptosis, but also during apoptosis and necrosis. Therefore, targeting NINJ1 could be a downstream therapeutic strategy that suppresses propagation of the cell death-associated inflammatory response regardless of its upstream mechanism (pyroptotic or otherwise).

We also need to be wary of the limitations in how we interpret the existing literature on pyroptosis in retinal disease. Because the essential role of gasdermin in pyroptosis was only recently established in 2015, earlier research on pyroptosis in retinal disease could only aim to identify inflammasome and caspase-1 activation in these diseases. We now know that activated inflammasomes can cause caspase-1 to cleave and release inflammatory cytokines without resulting in cell death (9, 28). Thus, we cannot assume that inflammasome activity, presence of mature caspase-1, and release of inflammatory cytokines in retinal cells necessarily means that pyroptosis is occurring in those cells. In addition, with the discovery that caspases 3 and 8 can activate GSDME and GSDMD (respectively) to mediate pyroptosis, we must also re-evaluate previous results suggesting that activation of these caspases in retinal cells represented apoptotic cell death. This also supports that there is significant overlap and a complex interplay between pyroptotic and apoptotic cell death that we do not currently understand. Many questions related to this require further study. For one, under what conditions do caspases 3 and 8 favor cleaving gasdermin over their usual apoptotic substrates? Furthermore, what other cell types and pathologies besides those already identified demonstrate caspase-3/8-mediated pyroptosis as opposed to the more well-known mechanisms of pyroptosis? GSDME-mediated pyroptosis is increasingly being demonstrated to play a role in cancer (94), but its involvement in neurodegenerative diseases including retinal diseases is still largely unexplored. There is also the question of if GSDMA/B/C-mediated pyroptosis has a role to play in retinal diseases. The gold standard for demonstrating the occurrence of pyroptosis should be the identification of cleaved N-terminal of gasdermin proteins in well-established models of retinal disease. More studies like this would provide a better foundation for us to determine if gasdermin-mediated therapy is a viable strategy for the treatment of retinal pathologies. Gasdermin-mediated therapies are currently being studied in tumor treatment (9), and potential translation of these therapies to retinal diseases is another area for future research.

## Author Contributions

MZ sourced and analyzed the referenced literature, conceived the structure of the manuscript, and wrote the manuscript. SL was a significant contributor in reviewing the drafts of the manuscript, adding to section “Pyroptosis and Glaucoma” of the manuscript, and creating supplementary figures. JM was a significant contributor in refining the topic of the review, conceiving the structure of the manuscript, reviewing the drafts of the manuscript, creating supplementary figures, and providing funding for the study. All authors have read and approved the final manuscript.

## Funding

Authors acknowledge funding from Canadian Institutes of Health Research, Natural Science and Engineering Research Council, and National Institutes of Health Research.

## Conflict of Interest

The authors declare that the research was conducted in the absence of any commercial or financial relationships that could be construed as a potential conflict of interest.

## Publisher's Note

All claims expressed in this article are solely those of the authors and do not necessarily represent those of their affiliated organizations, or those of the publisher, the editors and the reviewers. Any product that may be evaluated in this article, or claim that may be made by its manufacturer, is not guaranteed or endorsed by the publisher.
