# Supplementary material for: Targeting Pyroptotic Cell Death Pathways in Retinal Disease
Source: Front Med (Lausanne). 2022 Jan 3;8:802063. doi: 10.3389/fmed.2021.802063 (PMC8763245; doi:10.3389/fmed.2021.802063)
Supplement: Supplementary file 1 [file Data_Sheet_1.PDF]

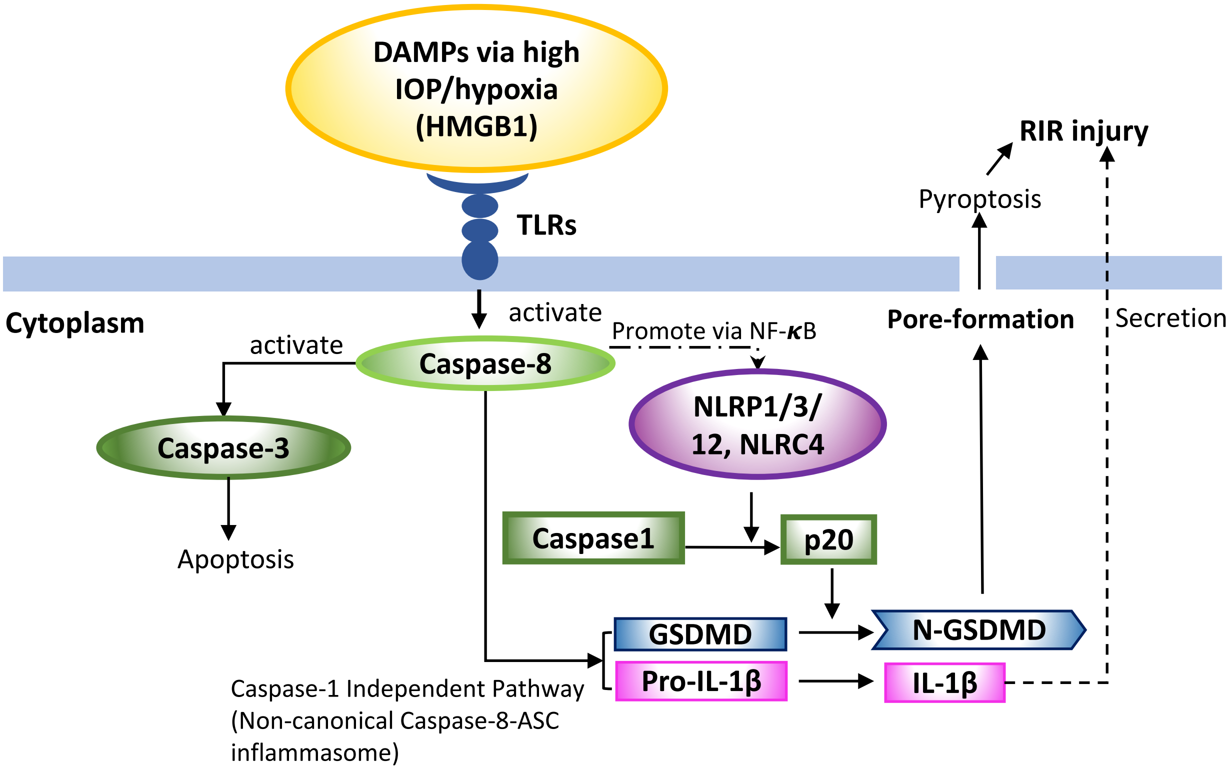

Supplementary Figure.1 Role of caspase-8 in acute glaucoma/retinal ischemia-reperfusion (RIR) injury. Caspase-8 not only activates caspase-3 in apoptotic pathways, but also mediates GSDMD cleavage and promotes NLR inflammasomes in non-apoptotic pathways. (Chi et al(2014,2015); Orning et al(2018); Cheng et al (2020)). DAMPs=danger-associated molecular patterns; HMGB1=high-mobility group box 1; TLRs=toll-like receptors; GSDMD=gasdermin D; NLRP1/3/12=nucleotide-binding and oligomerization domain (NOD)-like receptor family pyrin domain-containing 1/3/12; NLRC4=NLR family CARD domain-containing protein 4; RIR=retinal ischemia-reperfusion.
